# Ranking the Harm of Psychoactive Drugs Including Prescription Analgesics to Users and Others–A Perspective of German Addiction Medicine Experts

**DOI:** 10.3389/fpsyt.2020.592199

**Published:** 2020-10-26

**Authors:** Udo Bonnet, Michael Specka, Michael Soyka, Thomas Alberti, Stefan Bender, Torsten Grigoleit, Leopold Hermle, Jörg Hilger, Thomas Hillemacher, Thomas Kuhlmann, Jens Kuhn, Christian Luckhaus, Christel Lüdecke, Jens Reimer, Udo Schneider, Welf Schroeder, Markus Stuppe, Gerhard A. Wiesbeck, Norbert Wodarz, Heath McAnally, Norbert Scherbaum

**Affiliations:** ^1^Department of Psychiatry, Psychotherapy and Psychosomatic Medicine, Evangelisches Krankenhaus Castrop-Rauxel, Academic Teaching Hospital of the University of Duisburg/Essen, Castrop-Rauxel, Germany; ^2^Department of Psychiatry and Psychotherapy, Faculty of Medicine, Landschaftsverband Rheinland-Hospital Essen, University of Duisburg-Essen, Essen, Germany; ^3^Department of Psychiatry and Psychotherapy University Hospital, Ludwig-Maximilians-Universität München, Munich, Germany; ^4^Department of Psychiatry, Psychotherapy and Psychosomatic, Johanniter Hospital Oberhausen, Oberhausen, Germany; ^5^Psychiatry, Psychotherapy and Psychosomatics, Landschaftsverband Westfalen-Lippe-Hospital Marsberg, Marsberg, Germany; ^6^Psychiatry and Psychotherapy, Landschaftsverband Rheinland-Hospital Langenfeld, Langenfeld, Germany; ^7^Clinic for Psychiatry and Psychotherapy, Christophsbad, Göppingen, Germany; ^8^Clinic for Psychiatry, Psychotherapy, Psychosomatics and Neurology, Evangelische Stiftung Tannenhof, Remscheid, Germany; ^9^Department of Psychiatry, Socialpsychiatry and Psychotherapy, Hannover Medical School, Hanover, Germany; ^10^Department of Psychiatry and Psychotherapy, Paracelsus Medical University, Nuremberg, Germany; ^11^Clinic for Psychosomatics Bergisch-Gladbach, Bergisch Gladbach, Germany; ^12^Faculty of Medicine and University Hospital Cologne, University of Cologne, Cologne, Germany; ^13^Division of Cognitive Neuropsychiatry and Psychiatric Preventive Medicine, Department of Psychiatry, Landschaftsverband Westfalen-Lippe University Hospital Bochum, Ruhr University Bochum, Bochum, Germany; ^14^Lower Saxonian Psychiatric Hospital, Asklepios Hospital, Göttingen, Germany; ^15^Center for Interdisciplinary Addiction Medicine, University Medical Center Hamburg-Eppendorf, Hamburg, Germany; ^16^Health North Hospital Group Bemen, Bremen, Germany; ^17^Department of Psychiatry and Psychotherapy, Ruhr-University Bochum, Campus Ostwestfalen-Lippe, Luebbecke, Germany; ^18^MEDIAN Clinics Wied, Wied, Germany; ^19^Department of Addiction Medicine, Helios Medical Center Schwerin, Carl-Friedrich-Flemming-Clinic, Schwerin, Germany; ^20^Psychiatric Hospital, University of Basel, Basel, Switzerland; ^21^Department of Psychiatry and Psychotherapy, University of Regensburg, Regensburg, Germany; ^22^Northern Anesthesia & Pain Medicine, Limited Liability Company, Eagle River, AK, United States; ^23^Department of Anesthesiology and Pain Medicine, University of Washington School of Medicine, Seattle, WA, United States

**Keywords:** gabapentinoids, psychoropic drugs use, alcohol, illicit abused substance, new psychoactive drugs

## Abstract

**Background:** Over the past 15 years, comparative assessments of psychoactive substance harms to both users and others have been compiled by addiction experts. None of these rankings however have included synthetic cannabinoids or non-opioid prescription analgesics (NOAs, e.g., gabapentinoids) despite evidence of increasing recreational use. We present here an updated assessment by German addiction medicine experts, considering changing Western consumption trends–including those of NOAs.

**Methods:** In an initial survey, 101 German addiction medicine physicians evaluated both physical and psychosocial harms (in 5 dimensions) of 33 psychoactive substances including opioids and NOAs, to both users and others. In a second survey, 36 addiction medicine physicians estimated the relative weight of each health and social harm dimension to determine the overall harm rank of an individual substance. We compared our ranking with the most recent European assessment from 2014.

**Results:** Illicit drugs such as methamphetamine, heroin, cocaine and also alcohol were judged particularly harmful, and new psychoactive drugs (cathinones, synthetic cannabinoids) were ranked among the most harmful substances. Cannabis was ranked in the midrange, on par with benzodiazepines and ketamine—somewhat more favorable compared to the last European survey. Prescribed drugs including opioids (in contrast to the USA, Canada, and Australia) were judged less harmful. NOAs were at the bottom end of the ranking.

**Conclusion:** In Germany, alcohol and illicit drugs (including new psychoactive substances) continue to rank among the most harmful addictive substances in contrast to prescribed agents including opioid analgesics and NOAs. Current laws are incongruent with these harm rankings. This study is the first of its kind to include comparative harm rankings of several novel abused substances, both licit/prescribed and illicit.

## Key Points

Illicit drugs such as methamphetamine, heroin, and cocaine, and also alcohol were judged particularly harmful.
Prescribed drugs including opioids (in contrast to the USA, Canada and Australia) and non-opioid analgesics including gabapentinoids were judged less harmful.
Current laws are somewhat incongruent with these harm rankings.

## Introduction

Abuse of addictive psychoactive substances is characterized by negative health and social consequences not only for the user, but also for non-users in the community or society (1, 2). The DSM-5 has defined various specific substance-related dependence and addiction conditions (3), and ICD-10 coding reflects distinct mental and behavioral disorders related to alcohol, tobacco, opiates, cocaine, stimulants, hallucinogens, sedatives and hypnotics, cannabis and cannabinoids, and volatile solvents (4).

Over the past 15 years, the relative health and social harms potential of various addictive substances has been determined in England (5), the Netherlands (6), Scotland (7), France (8), and most recently in Australia (9) by medical and non-medical addiction experts. The average overall harm of various substances is usually reported in relative rankings, based upon multi-decision analyses (5, 9) or relying on “*ad-hoc*” assessments (6–8) using validated health and social dimensions (5). These rankings do not necessarily display congruence with legislative and law enforcement priorities in terms of relative regulation and control of substances, with alcohol being a prime example of dissonance between overall harms and control efforts (5–9). Nutt et al. were the first to demonstrate this incongruity (5).

In 2014, a group of 40 medical and non-medical addiction experts from 21 EU countries came to the same conclusion (10). This survey included 20 substances (10). In the interim, as in other Western countries, there have been shifting patterns of substance abuse trends as well as political framework conditions in Germany, especially

- Increasing abuse of methamphetamine mainly in regions bordering the Czech Republic (11–13).
- Increasing occurrence of new psychoactive substances (NPS), in particular a plethora of synthetic cannabinoids and stimulants (mostly cathinones) (12–14).
- Increasing fatal overdoses with heroin/morphine, opioid-containing, and non-opioid analgesics, synthetic opioids, narcotics, amphetamine, amphetamine derivatives, methamphetamine, and NPS, accompanied by a decrease in overdose deaths through opioid dependence treatment drugs such as methadone and buprenorphine (11, 15).
- Increasing availability of highly potent cannabis products with increased risk for psychosis and addiction (11, 13, 16, 17).
- Legalization of medicinal marijuana and cannabinoids for medical prescription (18).

Given these developments, we sought to update the assessment of the health and social harms from substances that are commonly misused in Germany and elsewhere and also of substances less frequently abused in our country, but already emerging (11, 12). In this context, synthetic cannabinoids (14) were included into harms rankings for the first time. We also included index surveys of harms rankings for propofol, an intravenous anesthetic (19), and some non-opioid analgesics (NOA), i.e., gabapentinoids, non-steroidal anti-inflammatory drugs (NSAIDs), flupirtine, and triptans (20–24). We decided to include NOAs together with opioid analgesics into our ratings because gabapentin and pregabalin (gabapentinoids) have recently entered the focus of addiction medicine. In the last decade, several pharmacovigilance databases, population-based studies and case reports have warned of their potential abuse liabilities and putative contribution to fatal overdoses especially in combination with opioids (22, 23). Even though NSAIDs are commonly thought to be non-addictive, there are recent case reports (25, 26) and epidemiologic (27, 28) as well as clinical data (24) that are raising some safety concerns about this traditional view. Other NOAs have also shown potential abuse and dependence liability e.g., flurpirtine (21) or triptans (20). Therefore, we felt it prudent to include the aforementioned NOAs for the first time in a study of this kind, too. This study is the first of its kind to include comparative harm rankings of several novel abused substances, both licit/prescribed and illicit.

## Methods

This cross-sectional questionnaire-study comprised two consecutive steps (survey 1 and survey 2, see below), in which quantitative questionnaires were distributed in written form among German addiction medicine experts. These experts were recruited at German addiction congresses and conferences. Additionally, the questionnaires were sent via email to 40 heads of German drug addiction treatment centers who were asked to distribute them in their zone of influence among other addiction medicine experts. Only those questionnaires which had been filled out by physicians who (i) were specialists, i.e., had extra expertise in at least one medical specialty and (ii) had been working longer than 5 years in tertiary care hospitals in the field of substance use disorders (SUD) treatment were included in the analysis. The experts' identity was kept anonymous with the exception of information about their age, gender, specialties, years of professional experience, years of work in tertiary care of SUD, and main focus of professional work (acute care or rehabilitation hospital) (Table 1).

**Table 1:** Participants' characteristics.

**Surveys**		**Cohort 1**	**Cohort 2**
		**(*n* = 101)**	**(*n* = 36)**
Age (years old)	Mean (SD)	49.8 (9.6)	52.9 (6.9)
	Median	50	55
Gender	Female (*n*, %)	26 (25.7%)	10 (27.8%)
	Male (*n*, %)	75 (74.3%)	26 (72.2%)
Years of professional experience	Mean (SD)	21.6 (9.5)	24.9 (8.2)
	Median	20	26
Years of tertiary care of SUD	Mean (SD)	16.3 (8.4)	17.6 (7.4)
	Median	15	16,5
Main focus of professional work	Acute care hospital (*n*, %)	76 (75.2%)	26 (72.2%)
	Rehabilitation hospital (*n*, %)	25 (24.8%)	10 (27.8%)

The first survey was conducted from March 2016 to September 2017 and assessed the average harm of 33 substances in in 5 dimensions (physical harm to users, psychological harm to users, social harm to users, physical and psychological harm to others, and social harm to others). As shown in Supplementary Figure 1, these dimensions were defined by 16 criteria, which have been validated in several studies of this type (5, 9, 10) (see Supplementary Materials—Methods Section). Overall harm to users and overall harm to others comprised 3 (physical, psychological, social) dimensions and 2 (physical & psychological, social) dimensions, respectively (for details see Supplementary Figure 1). The assessments were carried out using 5-point scales (from “not harmful” to “extremely harmful”).

The questionnaire was returned by 122 physicians and from those 101 were evaluated since 21 experts did not meet the inclusion criteria. The physicians were allowed to decide for themselves whether to rate a substance or not, and they were instructed to estimate their professional experience (“no/little” or “moderate” or “a lot”) with each substance they had rated. This information was needed to assess the validity of the ratings and to verify defined exclusion criteria, i.e., a substance with <60% ratings or more than 60% “no/little experience” ratings was excluded from further analysis. Consequently, the substances ayahuasca, khat, and kratom had to be excluded from the harm-evaluation (Supplementary Figures 2 and 3).

**Figure 1:**
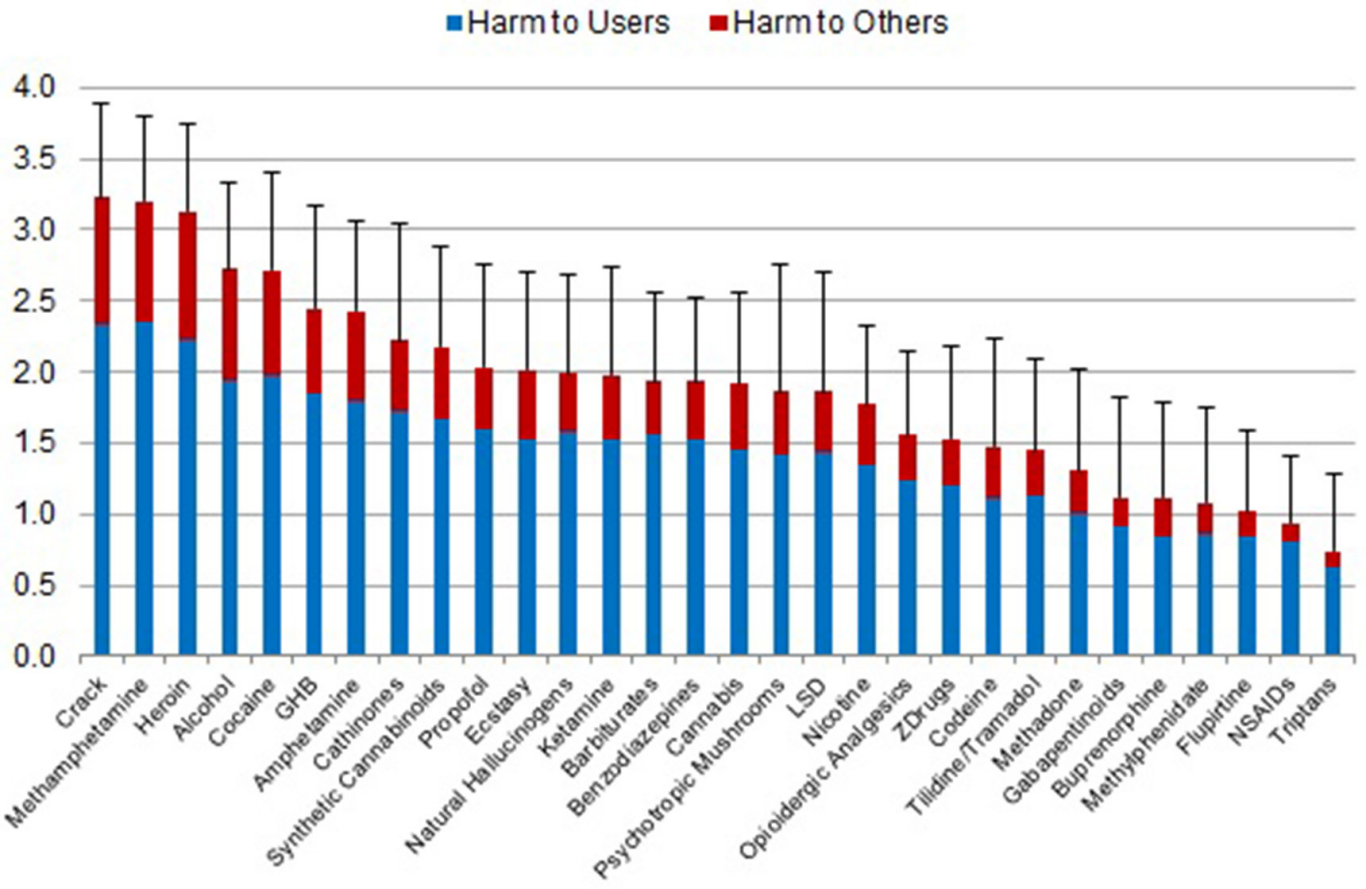
Average overall harm of 30 substances (mean values and standard deviations) as assessed by cohort 1 on a scale from 0 (‘not harmful’) to 4 (‘extremely harmful’), shown as harmful to users and harmful to others. The relative contribution of the 5 dimensions (Supplemental Figure 1, Supplemental Table 1) had been weighted by cohort 2.

The second survey (weighting of the dimensions to determine the overall harm in Figure 1) was conducted from September 2017 to May 2018 by cohort 2, which were recruited only from the emails to the aforementioned 40 heads of German drug addiction treatment centers. This follow-up survey was administered subsequently because the first survey was quite comprehensive, and combining the two surveys was deemed likely to overburden cohort 1 respondents, reducing the return quota. The second survey asked participants to estimate the relative weight (as a proportion between 0 and 1) of each of the 5 dimensions used in the first survey for the constitution of overall harm of psychotropic substances. All of the 36 returned questionnaires were included. We used the mean relative weight given by the 36 experts to each dimension for calculating the overall harm of each substance (Figure 1). Further details of the overall harm calculation of the remaining 30 substances and related data analyses including the comparison with the previous EU-ranking (Figure 3) are presented in the Supplementary Materials.

Validation of rankings was performed first by evaluating the magnitude of variability between the overall harm rating and any of the five component dimensions. A difference between the overall harm rating and any of the 5 separate ratings in the dimensions ≥8 ranks was considered significant and requires plausibility explanation (Table 2). An additional validation/sensitivity test was performed by substituting our survey-derived mean weights with the consensus-based weights of the previous EU-study (Supplementary Table 1) and comparing the resulting substance-ranks of Supplementary Figure 9 with those of Figure 1 (Supplementary Table 2).

**Table 2:** Plausibility check of the overall harm ranks.

**Substances/Rank in dimension**	**PHU**	**PSHU**	**SHU**	**PPHO**	**SHO**	**OH**	**LD–**	**LD+**
Crack	2	2	2	1	2	1	0	1
Methamphetamine	1	1	3	3	3	2	1	1
Heroin	5	4	1	2	1	3	2	2
Alcohol	4	8	5	4	4	4	0	4
Cocaine	7	3	4	5	5	5	2	2
GHB	6	5	7	7	7	6	1	1
Amphetamines	11	6	6	6	6	7	1	4
Cathinones	9	10	10	9	8	8	0	2
Synthetic cannabinoids	13	7	9	8	11	9	−2	3
Propofol	10	18	11	13	14	10	0	8
Ecstasy	15	16	12	10	9	11	−2	5
Natural hallucinogens	8	14	18	15	17	12	−4	5
Ketamine	14	15	13	11	12	13	−2	2
Barbiturates	12	12	17	19	20	14	−2	6
Benzodiazepines	16	9	15	18	16	15	−6	3
Cannabis	21	13	8	17	10	16	−8	5
Psychotropic mushrooms	18	17	16	14	13	17	−3	5
LSD	20	11	14	16	15	18	−7	2
Nicotine	3	25	24	12	18	19	−16	6
Opioidergic Analgesics	19	19	19	23	22	20	−1	3
ZDrugs	22	20	22	22	23	21	−1	2
Codeine	23	22	20	20	19	22	−3	1
Tilidine/Tramadol	24	21	21	21	24	23	−2	1
Methadone	26	24	23	24	21	24	−3	2
Gabapentinoids	27	23	27	27	27	25	−2	2
Buprenorphine	30	27	25	25	25	26	−1	4
Methylphenidate	28	26	26	26	26	27	−1	1
Flupirtine	26	28	28	28	28	28	−2	0
NSAIDs	17	29	29	29	29	29	−12	0
Triptans	29	30	30	30	30	30	−1	0

*The lower the largest difference (LD)-value the lower the variability of the 5 dimension-ranks with reference to the (individual) overall harm (OH)-rank of any substance. Discrepancies of ≥8 ranks are marked with grey horizontal background indicating considerable variability of the single dimension-ranks with reference to the individual OH-rank requiring plausible explanations. Abbreviations of the single dimensions: PHU – physical harm to users, PSHU – psychological harm to users, SHU – social harm to users, PPHO – physical & psychological harm to others, SHO – social harm to others, OH – overall harm, LD− – largest difference between OH-rank and any lower dimension-rank, LD+ – largest difference between OH-rank and any higher dimension-rank*.

## Results

### Sample and Participants' Experience

The specialist physicians had worked for a median of 15 years (cohort 1) and 16.5 years (cohort 2) in the tertiary care of patients with SUD. Approximately three out of four participants worked in acute care hospitals, with the remainder working in rehabilitation clinics (Table 1).

### Average Overall Harm

Experts' ratings in the 5 separate dimensions are shown in the (Supplementary Figures 4–8). Regarding overall harm, traditional drugs of abuse, i.e., cocaine (including “crack”), methamphetamine, heroin, and alcohol were ranked as being most harmful. The NPS, i.e., cathinones and synthetic cannabinoids, had subordinate positions in the top harm-level group. Ketamine, benzodiazepines, cannabis, psychotropic mushrooms, LSD, nicotine, and opioid analgesics were in the midrange. Methadone and buprenorphine (both preferred in Germany for maintenance therapy of opioid dependence) fell into the lower ranges, while methylphenidate (in Germany the preferred medication for ADHD-treatment), and NOAs were at the lowest ranges of the harm-ranking. Among the NOAs, gabapentin and pregabalin (gabapentinoids) were regarded as more harmful than flupirtine, NSAIDs and triptans (Figure 1).

### Difference Between Acute and Rehabilitation Hospital Raters?

**Figure 2:**
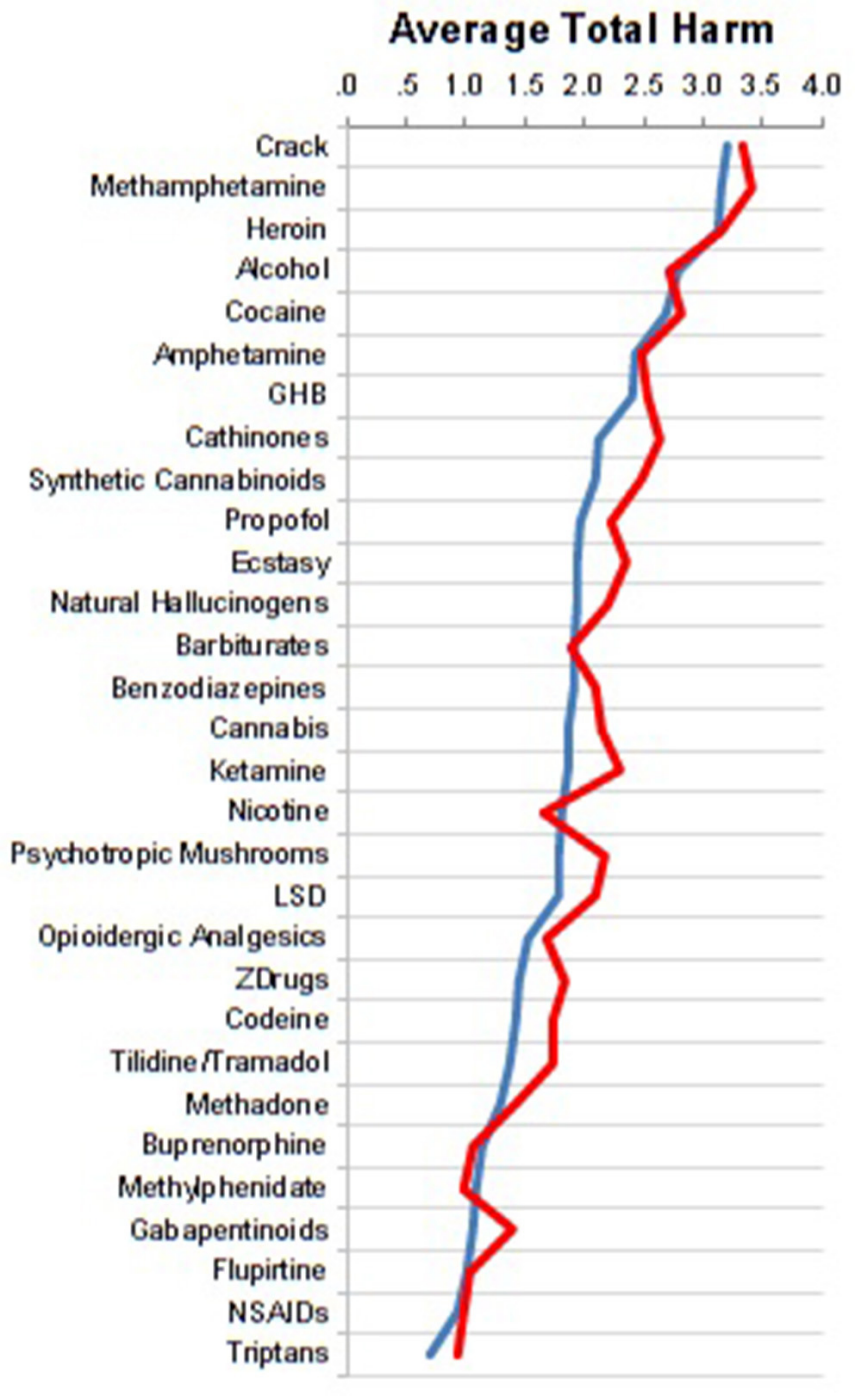
Comparison of assessments between specialists at acute (*n* = 76, blue curve) vs. rehabilitation hospitals (*n* = 25, red curve).

The assessments of the specialists from acute and rehabilitation hospitals were very similar as shown in Figure 2.

### Comparison With the Last European Analysis

**Figure 3:**
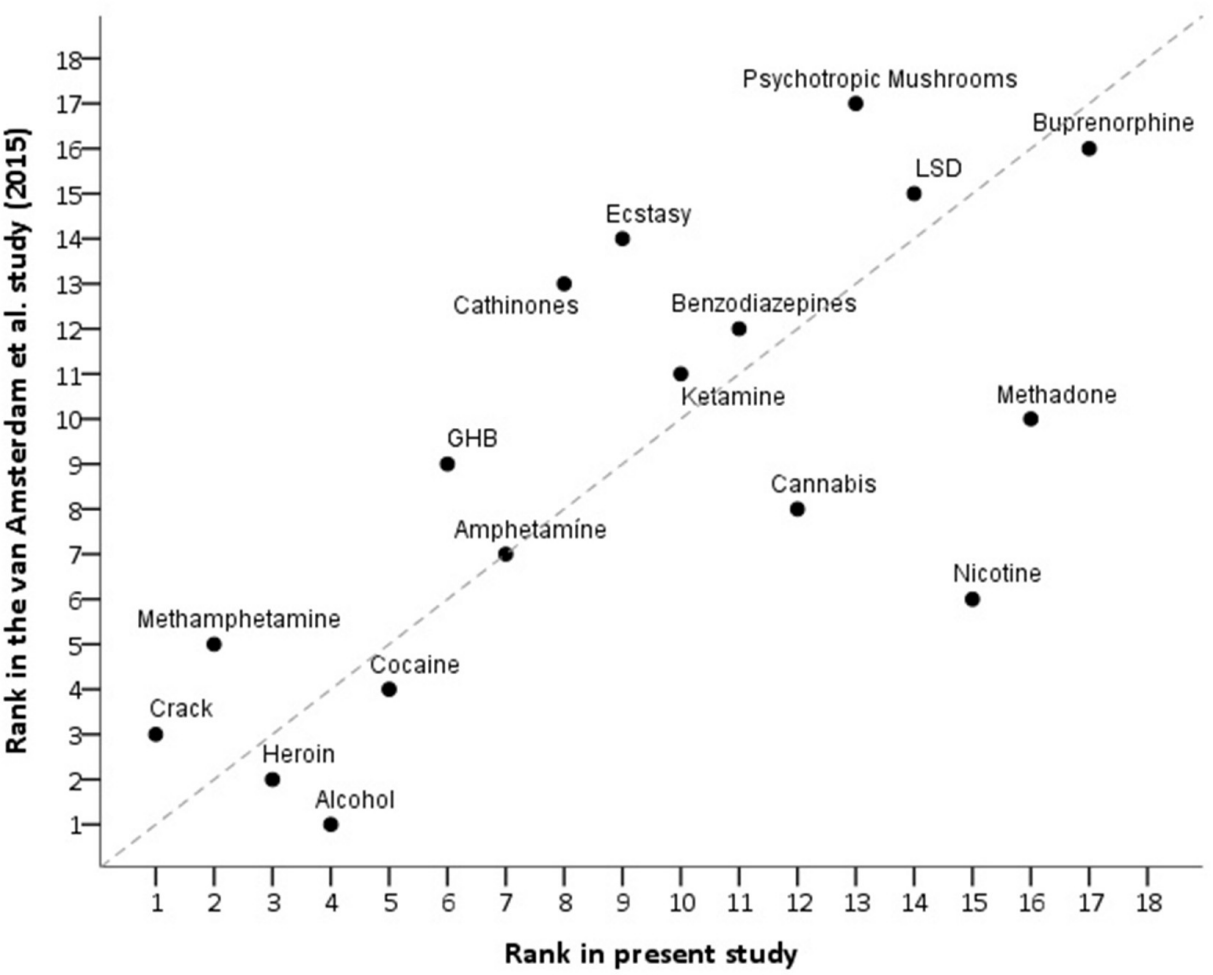
Correlation of the present assessment and the last EU-assessment (10) of the overall harm of drugs of abuse (rs = 0.73). For better orientation, the bisector indicates perfect correlation (rs = 1).

This updated German survey assessed methadone, nicotine, cannabis and alcohol as less harmful than did the EU-raters in 2014 (10), while psychotropic mushrooms, cathinones, ecstasy, GHB, methamphetamine, and crack were judged to be more harmful—see Figure 3.

### Plausibility Check and Sensitivity Test

The lowest discrepancies between the average overall harm-rank and the 5 health and social dimension-ranks were found for the traditional illegal drugs crack (and other cocaine), heroin, methamphetamine, and also for alcohol, which were also ranked at the top positions in terms of harms. The same applied to GHB and NPS ranking near the top, ketamine in the midrange, opioids at lower ranges, and most NOAs (gabapentinoids, flupirtine, triptans) at the lowest ranks. Striking discrepancies were seen for propofol, cannabis, nicotine and NSAIDs (Table 2). In case of nicotine and NSAIDs disproportionate physical harm concerns (e.g., cancer, stroke, coronary disease, COPD for the former, and GI bleeds, renal and cardiovascular disease for the latter) likely account for most of the discrepancy for those substances. In the case of cannabis, the German literature currently reflects a general perception of relatively low physical harms and conversely a perception of elevated psychosocial harms to users, which dichotomy serves to corroborate the discrepancy here (29–31). The discrepancy for nicotine (and perhaps also for propofol to some extent) may be owing in part to an unexpectedly low ranking of psychological harm to users which diverges from empiric evidence. This potential underestimation may therefore threaten the validity of the overall harm-ranks of these specific substances.

When alternatively, we used the consensus-based weights of the EU-rating study (10) as a comparison sensitivity test, we found that the resulting ranking of overall harms (Supplementary Figure 9) was very similar to our survey-derived weighted rankings shown in Figure 1 (see Supplementary Table 2 for comparison). This suggests that the outlier/skewed weightings of individual dimensions (Supplementary Table 1) do not critically influence the resulting overall harm rankings in our study.

## Discussion

Our data corroborate the situation in many other countries (5–10) of discordance between expert harm rankings of popular drugs of abuse and their regulation by narcotic laws, as evidenced most strikingly by the assessment of alcohol—judged to be among the most harmful substances abused in our country. The relatively high prevalence of alcohol use/abuse (compared to that of less-frequently abused but perhaps more dangerous substances) likely contributes to its dimension-specific ratings, e.g., harm to others, as well as to its overall position. Similarly, the decreasing prevalence of nicotine use in Germany (as tobacco smoking has been banned from many public areas such as hospitals, educational establishments, public transport, restaurants, pubs, and discos during the last 10 years or so) may contribute to a lower-than-expected harm ranking. In addition it should be mentioned that nicotine use, despite its ability to produce considerable behavioral dependence is hardly associated with dramatic psychiatric effects, e.g., in contrast to alcohol or hallucinogen use. This study was the first to compare the harms of various NOAs with harms of well-characterized substances of abuse, and as expected identifies the harms of NOAs to be considerably lower than those of the traditional substances of abuse. The present study was also the first to include synthetic cannabinoids and propofol in an overall-harm ranking schema, which may be beneficial for the psychoeducation of users, for regulatory considerations, or for defining fields of political action for health promotion.

NPS (cathinones and synthetic cannabinoids) have been assigned to the top harm-level group here. Policy-makers and clinicians would benefit from further data about the NPS-phenomenon, e.g., associated morbidity (32, 33) and mortality which are on the rise (33).

Compared with the EU-rating from 2014 (10), cannabis, methadone and nicotine were assessed as less harmful, while crack, methamphetamine, GHB, cathinones, ecstasy, and psychotropic mushrooms were seen as more harmful (Figure 3). Cannabis and hallucinogens (i.e., ketamine, psychotropic mushrooms and LSD) were considered to be on the harm level of benzodiazepines or barbiturates. It should be mentioned that psilocybin (in Figure 1 listed as psychotropic mushrooms) and LSD have both enjoyed re-emerging therapeutic potential in psychiatric diseases and appear to show low abuse potential in that context (34).

It is interesting to note that opioid analgesics were not within the top ranks of harmful drugs. This could perhaps be related to the fact that an “opioid epidemic” (such as that in the USA, Canada and Australia), is yet not apparent in Germany or in Western Europe (35–38). The relatively low harm rankings of prescription opioids in our study stand in stark contrast to the high level of stigmatization of illicit opioids. These findings are congruent with the multi-decision analysis of nine experts (8 from the United Kingdom and 1 from the Netherlands) suggesting that the overall harms of non-medically used prescription opioids are less than half that of injected street heroin (39).

Methadone was assessed as less harmful than standard opioid analgesics, which viewpoint might be biased by addiction medicine physicians' conception of methadone primarily as a standard opioid dependence maintenance treatment, which in this context has been repeatedly shown to reduce morbidity and mortality (15). In the context of illicit use and abuse, methadone's harms (e.g., apneic and torsades-de-pointe deaths, addiction, and diversion) are obviously considerably higher than those of several other drugs ranked above it. This exposes a major limitation of drug harm-ranking studies based upon subjective assessments as they may not allow for clear differentiation between the harms of a drug with therapeutic indication in a medical context vs. illicit use/misuse outside of that context. These discrepancies in ranking of analgesics among other agents suggest that perhaps raters' experience in pain medicine should have been surveyed as well.

It cannot be excluded that our ratings may be biased toward metropolitan rather than rural perception of substance use harms; clarifying this would require further study in larger samples. Also, a possible gender influence on drug harm perceptions was not explicitly investigated here (40, 41). As we had sent out the questionnaires without tracking all recipients, requesting forwarding to other German addiction medicine experts, we are unable to provide information about the exact number of experts who finally received our questionnaires. However, such modus operandi is not unusual for studies of this kind (5). Other limitations, similar to previous studies (5–10) include the fact that the present work cannot claim to meet strict requirements for representativeness. We aimed to reduce subjectivity biases by recruiting a large and homogeneous study group (all physicians specializing in addiction medicine). However, no official statistic exists for how many specialists with more than 5 years of experience in tertiary care of SUD were working in Germany at the time of the study. We estimate that number to be somewhere between 250 and 500 physicians, thus our sample may yield a minority viewpoint. In Germany, addiction medicine experts usually are psychiatrists or general practitioners. Unlike the English (5), EU (10) and Australian (9) studies, we used no consensus–feedbacks. While this additional step may have increased the likelihood of survey participants' agreement (42), we decided against this course, because consensus-based decisions *per se* do not eliminate subjectivity (43) and there exists no “one-size-fits-all-method” for benefit-risk assessment (44). Furthermore, prior consensus-based studies utilized smaller samples comprising addiction experts from different professions (5, 9, 10), whose heterogeneity of experiences in the treatment of SUD more likely needed a consensus-based decision strategy than did our homogeneous group. Similar to the Netherlands (6), the Scottish (7), and the French research groups (8) we performed an “*ad-hoc*” assessment, using validated health and social dimensions, which have been utilized in previous (5, 10) and recent (9) empirical studies. This decision to use an “*ad-hoc*” format maximized the return of completed questionnaires.

Apart from the novel inclusion of NOAs, synthetic cannabinoids and propofol, there are a few strengths of the present study: (i) the utilization of one of the largest samples in this type of study; (ii) the considerable multidimensional addiction medicine experience of the participants, including that of rehabilitation clinic specialists (Figure 2), which in Germany focuses heavily upon psychosocial dimensions and outcomes; (iii) comparison with the previous EU-rating (Figure 3); and (iv) the addition of comparisons of illicit and licit drug rankings to the current literature.

The results of this cross-sectional questionnaire-study update the average overall harm (with component harms from various health and social dimensions) arising from use/misuse of various psychoactive substances (including prescription analgesics) from the perspective of German addiction medicine specialists. It should be emphasized however that these relative overall rankings apply to population-level risks, and depending on the individual and situational context as well as on the intensity of the individual misuse, nearly every psychoactive substance can be used in a very dangerous and harmful way.

## Conclusion

This study provides an updated German addiction medicine expert ranking of the average overall harms as well as harms in specific health and social dimensions of various psychoactive substances, including analgesics. Alcohol was estimated to be among the most harmful addictive substances, along with heroin, cocaine, methamphetamine, GHB, and NPS (i.e., synthetic cannabinoids, cathinones). The elevated risks of alcohol are somewhat discordant with the German narcotic law, similar to most countries. Cannabis and ketamine were ranked in midrange on par with benzodiazepines. Therapeutically used drugs such as non-opioid analgesics, methylphenidate, and opioids were estimated to be on the whole to be the least harmful at present. Such relative safety perception however is certainly subject to change should misuse and abuse patterns change over time (45).

## Data Availability Statement

The raw data supporting the conclusions of this article will be made available by the authors, without undue reservation.

## Ethics Statement

The studies involving human participants were reviewed and approved by Ethik-Kommission der Medizinischen Fakultät der Universität Duisburg-Essen. Written informed consent for participation was not required for this study in accordance with the national legislation and the institutional requirements.

## Author Contributions

UB: conception and design. MSp: analysis of the data. UB and MSp: collection and interpretation of data. UB: drafting the article. All authors: revising it critically for important intellectual content.

## Conflict of Interest

NS has received honoraria for several activities (e.g., advisory board membership, lectures, manuscripts) from AbbVie, Camurus, Hexal, Janssen-Cilag, MSD, Medice, Mundipharma, Reckitt-Benckiser/Indivior, and Sanofi-Aventis. During the last 3 years he has participated in clinical trials financed by the pharmaceutical industry. TA has received honoraria (e.g., advisory board membership) and/or educational grants from Janssen-Cilag, Medice, and Otsuka-Lundbeck NW has received honoraria for (not product-related) lectures (Janssen-Cilag, mundipharma, and Reckitt-Benckiser/Indivior), During the last 3 years he has participated in clinical trials financed by the pharmaceutical industry and received public funding (BayStMGP) for the evaluation of Take-Home Naloxone. TH has received honoraria for several activities (e.g., advisory board membership, lectures) from Janssen-Cilag, Amomed, Shire, Takeda, Servier MSo has been working as a consultant or has Received speakers freut from Ammomed, Indivior, Camurus for the past 3 years. JR has received honoraria for participation in advisory boards, consulting and lectures from AbbVie, Camurus, Gilead, Hexal, Indivior, and Sanofi-Aventis. JK has received honoraria from Bayer, Janssen, Lundbeck, Neuraxpharm, Otsuka Pharma, Schwabe, and Servier for lecturing at conferences and financial support to travel. He has received financial support for Investigator initiated trials from Medtronic GmbH. HM is also affiliated with a private praxis (Northern Anesthesia; Pain Medicine, LLC, Eagle River, AK, USA), which has no bearing on this study. The remaining authors declare that the research was conducted in the absence of any commercial or financial relationships that could be construed as a potential conflict of interest.
