# Supplementary material for: Ranking the Harm of Psychoactive Drugs Including Prescription Analgesics to Users and Others–A Perspective of German Addiction Medicine Experts
Source: Front Psychiatry. 2020 Oct 26;11:592199. doi: 10.3389/fpsyt.2020.592199 (PMC7649209; doi:10.3389/fpsyt.2020.592199)
Supplement: Supplementary file 1 [file Data_Sheet_1.pdf]

Supplementary Materials

Ranking the harm of psychoactive drugs including prescription analgesics to users and others - A perspective of German addiction medicine experts

by

Udo Bonnet, Michael Specka, Michael Soyka, Thomas Alberti, Stefan Bender, Torsten Grigoleit, Leopold Hermle, Jörg Hilger, Thomas Hillemacher, Thomas Kuhlmann, Jens Kuhn,´Christian Luckhaus, Christel Lüdecke, Jens Reimer, Udo Schneider, Welf Schroeder, Markus Stuppe, Gerhard A. Wiesbeck, Norbert Wodarz, Heath McAnally, Norbert Scherbaum

Correspondence

Udo Bonnet, Klinik für Psychiatrie, Psychotherapie und Psychosomatik Evangelisches Krankenhaus Castrop-Rauxel, 44577 Castrop-Rauxel, Grutholzallee 21, Email: udo.bonnet@uni-due.de

Supplementary Materials

1. Methods

Based upon initial research of and European group, who had used a Multi Criteria Decision Analysis (MCDA) [10], we developed a questionnaire to estimate the harm of an addictive drug in 5 health and social dimensions, whose structures are is shown in Supplemental Figure 1.

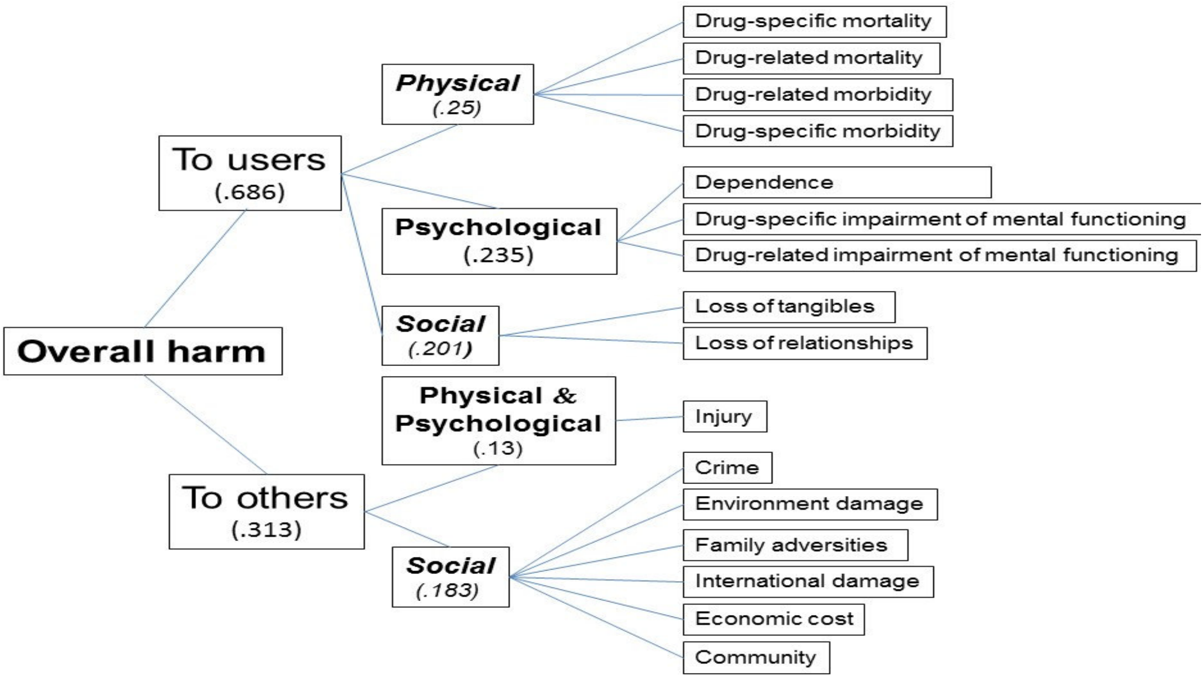

Supplemental Figure 1: Structure of the questionnaire and its evaluation. For each substance, in a first step, per 5-point scale (from "not harmful" to "extremely harmful") a sum for every 5 physical, psychological, and social dimension (bold letters) being assigned to harms to users and harms to others was determined by cohort 1. These dimensions had been defined by the 16 criteria usually analyzed in studies of this type (all boxes on the right). In a second step, these 5 dimensions were weighted according to their relative harm-relevance for addictive agents (in brackets) by cohort 2. The result of step 1 for every dimension of a substance was multiplied with the weight of this dimension. The results of all 5 dimensions were summed up to the overall harm of the substance under study.

We also used the weightings of the 5 health and social dimensions of the previous EU-rating [10] which differ from our assessment (Supplemental Table 1). As we put more weight on psychological and social harm to the user, and less weight on social harm

to others, our overall harm calculation with the EU-weights instead of the survey-based weights (of cohort 2) should serve as a sensitivity test.

Supplemental Table 1: weights of the different dimensions of harm of addictive substances based upon the same criteria

Dimension	*EU-rating**	Present study** Mean (SD) in %
Physical harm to user	25,9	25,0 (7,0)
Psychological harm to user	16,0	*23,5 (6,6)*
Social harm to user	11,2	*20,1 (7,5)*
Physical & psychological harm to others	11,6	13,0 (4,4)
Social harm to others	35,5	*18,3 (7,7)*

*consensus-based [10], **ad hoc, clearly different estimations are marked by grey background.

As we intended to receive as many as possible completed questionnaires in survey 1 we calculated to receive approximately one third of those returning for the subsequent survey 2. De facto we received 36 completed questionnaires in this second survey which was in the magnitude of most previous studies of this type [5,6,8-10].

Using the EU-weights the average overall harm was determined as follows: Overall harm = Physical harm to user x 0.259 + Psychological harm to user x 0.16 + Social harm to the user x 0.112 + Physical and psychological harm to others x 0.116 + Social harm to others x 0.335 (Supplemental Table 1, Supplemental Figure 9). The same was done with our weights from survey 2 (Supplemental Table 1, Supplemental Figure 1) to generate the data shown in Figure 1: Overall harm = Physical harm to user x 0.25 + Psychological harm to user x 0.235 + Social harm to user x 0.201 + Physical and psychological harm to others x 0.13 + Social harm to others x 0.183 (Supplemental Table 1, Supplemental Figure 9).

Data analysis was descriptive. For comparison of our ranking with that of the EU-group (Figure 3) we used Spearman´s rho (rs).

1.1. Exclusion criteria

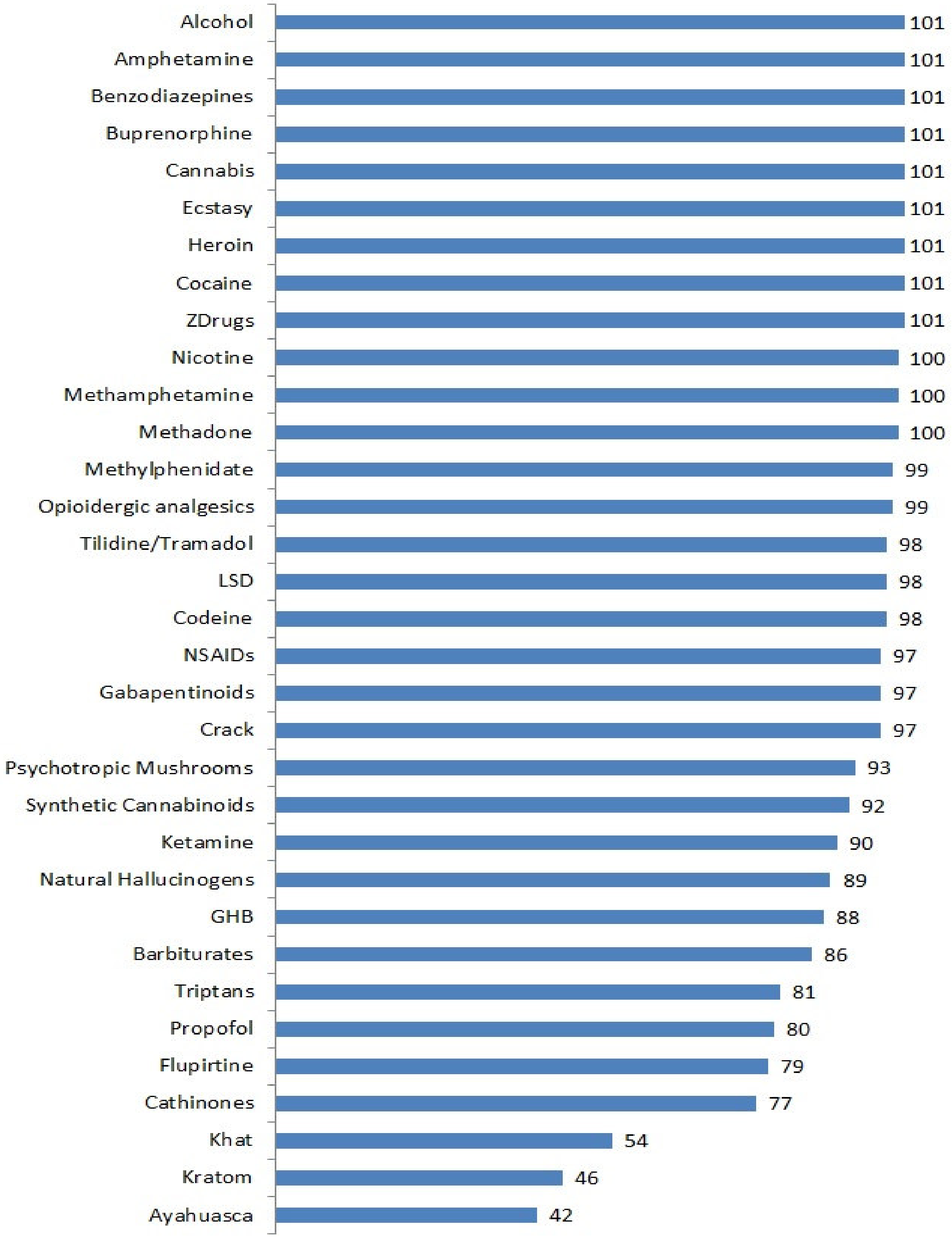

Supplemental Figure 2: Number of assessments made per substance by cohort 1. For data analysis, the substances khat, kratom and ayahuasca were excluded because less than 60% of the questions about these substances had been returned.

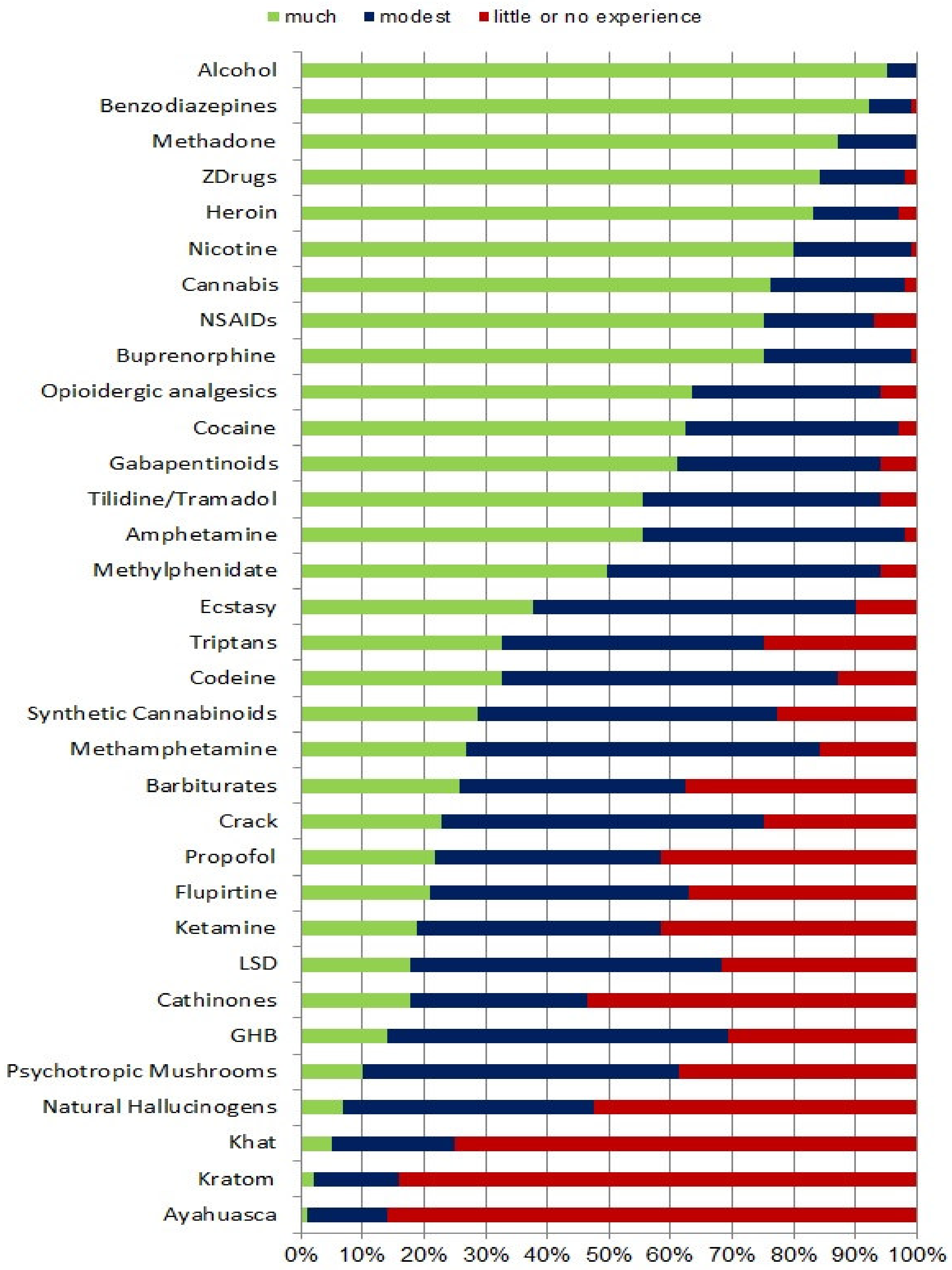

Supplemental Figure 3: Raters´ clinical experience regarding the individual substances in cohort 1. For data analysis, khat, kratom and ayahuasca were excluded, since more than 60% of the responses indicated no / little experience with these substances.

2. Results

2.1. Assessment of the average substance harm in the 5 separate health and social dimensions

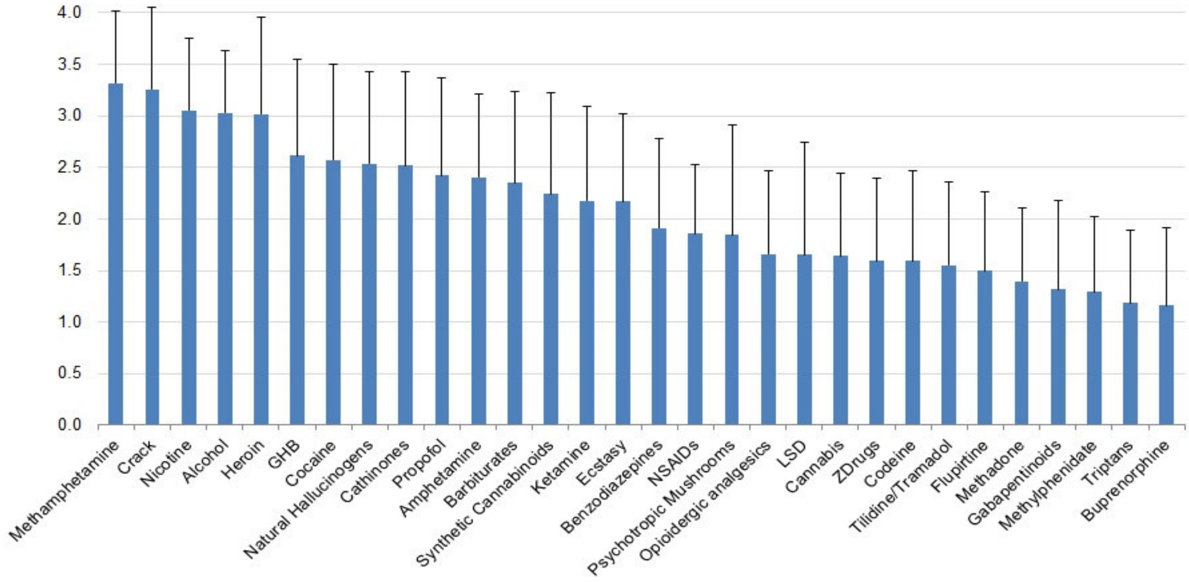

Supplemental Figure 4: Mean (SD) of the 30 substances in the dimension **physical harm to users** on a scale from 0 "not harmful" to 4 "extremely harmful.

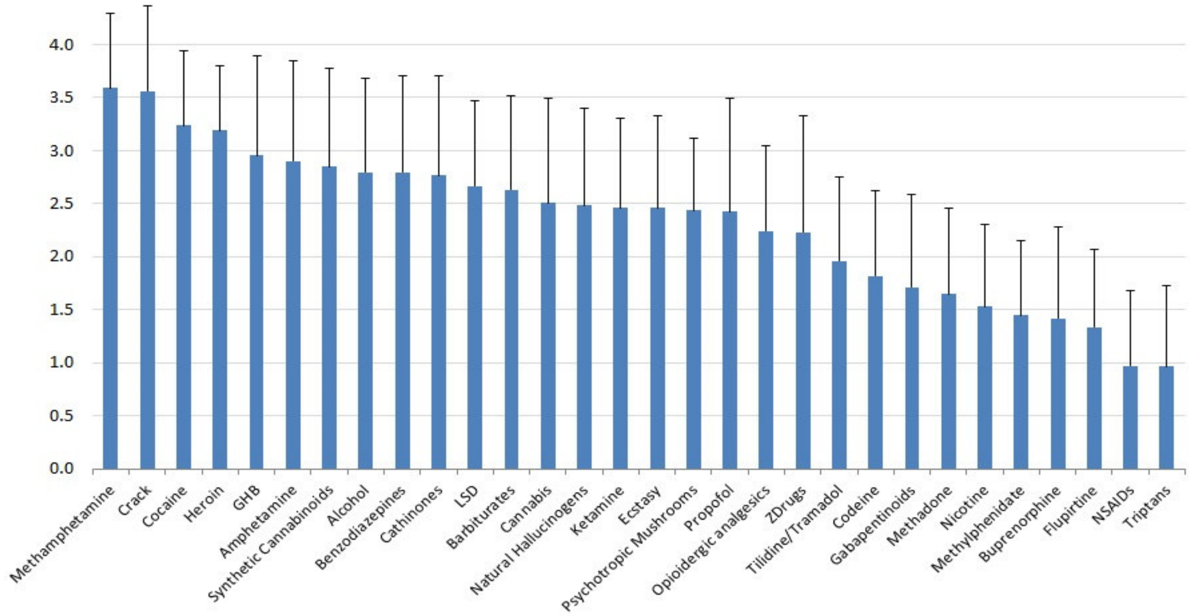

Supplemental Figure 5: Mean (SD) of the 30 substances in the dimension **psychological harm to users** on a scale from 0 "not harmful" to 4 "extremely harmful.

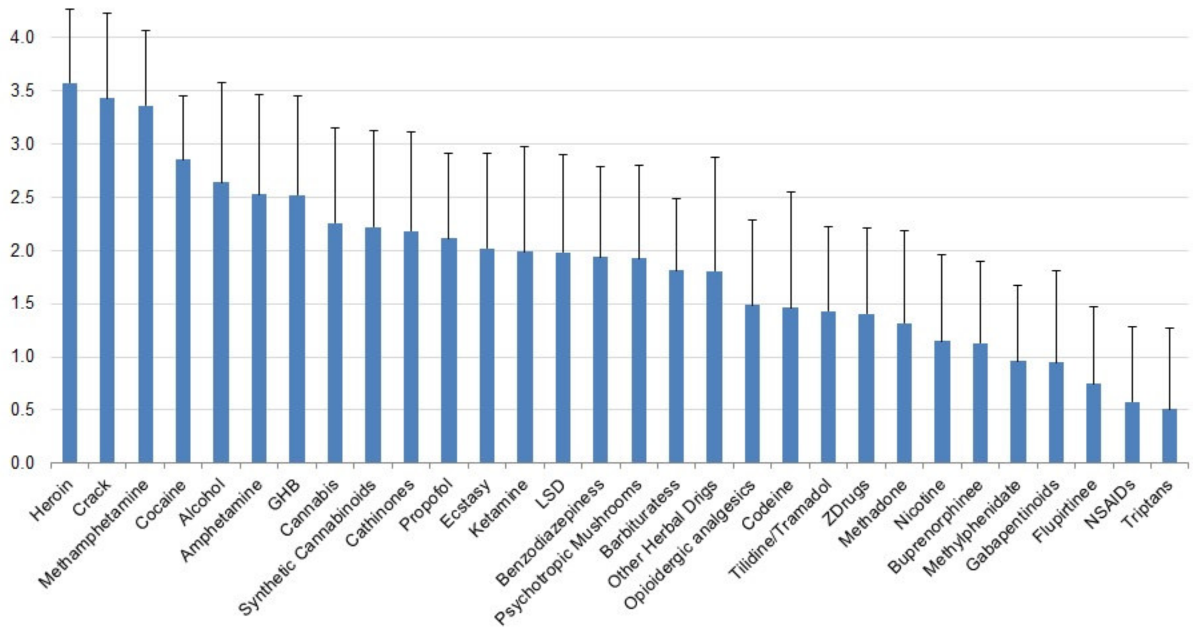

Supplemental Figure 6: Mean (SD) of the 30 substances in the dimension **social harm to users** on a scale from 0 "not harmful" to 4 "extremely harmful.

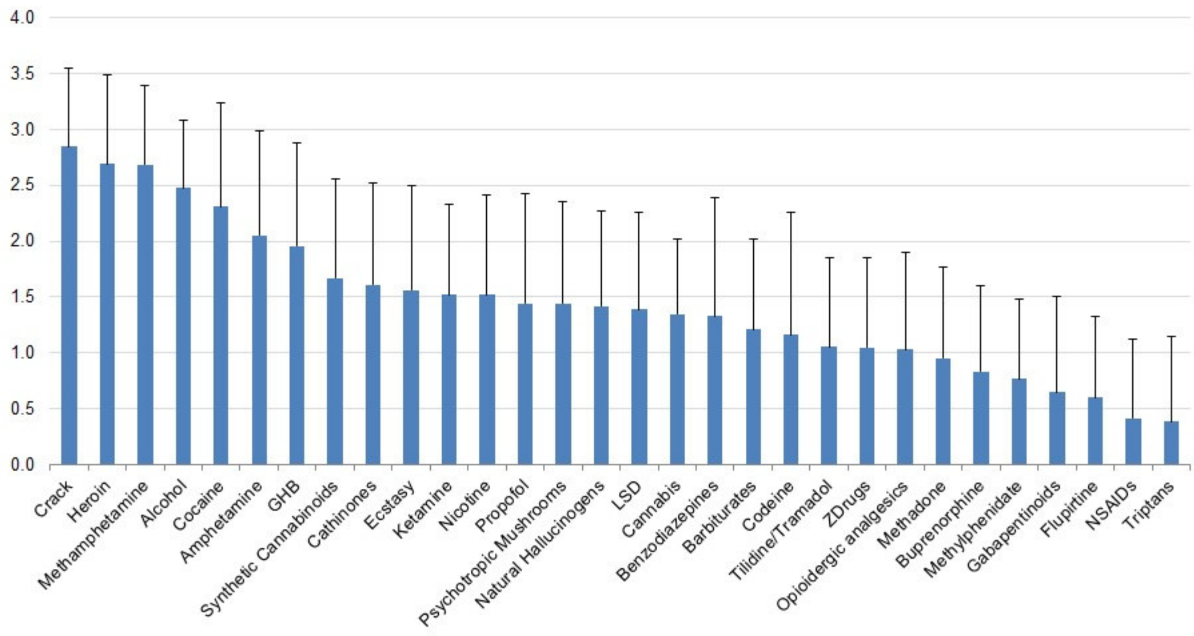

Supplemental Figure 7: Mean (SD) of the 30 substances in the dimension **physical & psychological harm to others** on a scale from 0 "not harmful" to 4 "extremely harmful.

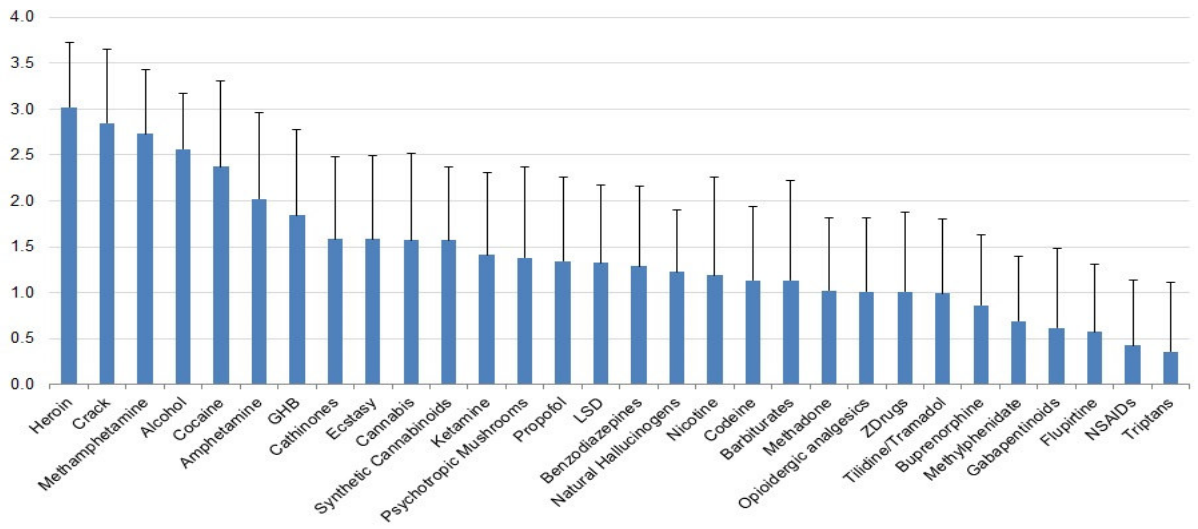

Supplemental Figure 8: Mean (SD) of the 30 substances in the dimension **social harm to others** on a scale from 0 "not harmful" to 4 "extremely harmful.

2.2. Overall harm – Sensitivity Test

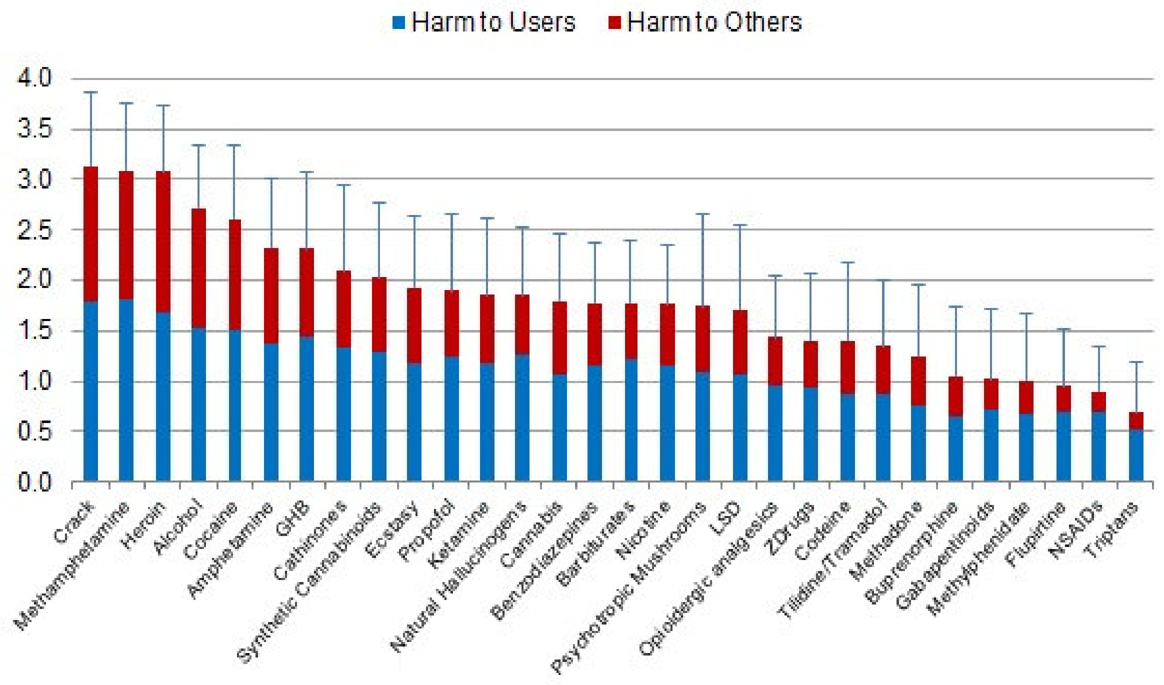

Supplemental Figure 9 (sensitivity test): Mean (SD) of the overall harm of the 30 substances **using the weights of the EU-rating** [10] on a scale from 0 "not harmful" to 4 "extremely harmful. The ranks are very similar to the ranks of Figure 1 produced with our weights (see Supplemental Table 2).

Supplemental Table 2: Comparison of the ranks of an individual substance between Figure 1* and Supplemental Figure 9**

Substance	Rank in Figure 1 (Rank A)	Rank in Supplemental Figure 9 (Rank B)	Difference between Rank A and Rank B
Crack	1	1	0
Methamphetamine	2	2	0
Heroin	3	3	0
Alcohol	4	4	0
Cocaine	5	5	0
GHB	6	7	-1
Amphetamine	7	6	1
Cathinones	8	8	0
Synthetic Cannabinoids	9	9	0
Propofol	10	11	-1
Ecstasy	11	10	1
Natural Hallucinogens	12	13	.1
Ketamine	13	12	1
Barbiturates	14	16	-2
Benzodiazepines	15	15	0
Cannabis	16	14	2
Psychotropic Mushrooms	17	18	-1
LSD	18	19	-1
Nicotine	19	17	2
Opioidergic Analgesics	20	20	0

Z-Drugs	21	21	0
Codeine	22	22	0
Tilidine/Tramadol	23	23	0
Methadone	24	24	0
Gabapentinoids	25	25	0
Buprenorphine	26	26	0
Methylphenidate	27	27	0
Flupirtine	28	28	0
NSAIDs	29	29	0
Triptans	30	30	0

*using survey-based weights of cohort 2 (Table1)

**using weights of the EU-rating [10]

Literature

according to the main article
